# Simple clinical and laboratory predictors to improve empirical treatment strategies in areas of high scrub typhus and dengue endemicity, central Vietnam

**DOI:** 10.1371/journal.pntd.0010281

**Published:** 2022-05-04

**Authors:** Hanh Thi Duc Tran, Christian Schindler, Thuy Thi Thanh Pham, Mai Quang Vien, Hung Manh Do, Quyet Thi Ngo, Trieu Bao Nguyen, Hang Thi Hai Hoang, Lan Thi Hoang Vu, Esther Schelling, Daniel H. Paris

**Affiliations:** 1 Department of Epidemiology and Public Health, Swiss Tropical and Public Health Institute, Basel, Switzerland; 2 Department of Medicine, Swiss Tropical and Public Health Institute, Basel, Switzerland; 3 Department of Epidemiology, Hanoi University of Public Health, Hanoi, Vietnam; 4 University of Basel, Basel, Switzerland; 5 Department of Infectious Diseases, Bach Mai Hospital, Hanoi, Vietnam; 6 The Partnership for Health Advancement in Vietnam (HAIVN), Hanoi, Vietnam; 7 Nha Trang Pasteur Institute, Khanh Hoa, Vietnam; 8 Department for Infectious Disease Control and Prevention, Nha Trang Pasteur Institute, Khanh Hoa, Vietnam; 9 Department of Microbiology and Immunology, Nha Trang Pasteur Institute, Khanh Hoa, Vietnam; 10 Vétérinaires Sans Frontières Suisse, Bern, Switzerland; University of Peradeniya Faculty of Medicine, SRI LANKA

## Abstract

**Background:**

Dengue fever is highly endemic in Vietnam, but scrub typhus—although recognized as an endemic disease—remains underappreciated. These diseases together are likely to account for more than half of the acute undifferentiated fever burden in Vietnam. Scrub typhus (ST) is a bacterial disease requiring antimicrobial treatment, while dengue fever (DF) is of viral etiology and does not. The access to adequate diagnostics and the current understanding of empirical treatment strategies for both illnesses remain limited. In this study we aimed to contribute to the clinical decision process in the management of these two important etiologies of febrile illness in Vietnam.

**Methods:**

Using retrospective data from 221 PCR-confirmed scrub typhus cases and 387 NS1 protein positive dengue fever patients admitted to five hospitals in Khanh Hoa province (central Vietnam), we defined predictive characteristics for both diseases that support simple clinical decision making with potential to inform decision algorithms in future. We developed models to discriminate scrub typhus from dengue fever using multivariable logistic regression (M-LR) and classification and regression trees (CART). Regression trees were developed for the entire data set initially and pruned, based on cross-validation. Regression models were developed in a training data set involving 60% of the total sample and validated in the complementary subsample. Probability cut points for the distinction between scrub typhus and dengue fever were chosen to maximise the sum of sensitivity and specificity.

**Results:**

Using M-LR, following seven predictors were identified, that reliably differentiate ST from DF; eschar, regional lymphadenopathy, an occupation in nature, increased days of fever on admission, increased neutrophil count, decreased ratio of neutrophils/lymphocytes, and age over 40. Sensitivity and specificity of predictions based on these seven factors reached 93.7% and 99.5%, respectively. When excluding the “eschar” variable, the values dropped to 76.3% and 92.3%, respectively.

The CART model generated one further variable; increased days of fever on admission, when eschar was included, the sensitivity and specificity was 95% and 96.9%, respectively. The model without eschar involved the following six variables; regional lymphadenopathy, increased days of fever on admission, increased neutrophil count, increased lymphocyte count, platelet count ≥ 47 G/L and age over 28 years as predictors of ST and provided a sensitivity of 77.4% and a specificity of 90.7%.

**Conclusions:**

The generated algorithms contribute to differentiating scrub typhus from dengue fever using basic clinical and laboratory parameters, supporting clinical decision making in areas where dengue and scrub typhus are co-endemic in Vietnam.

## Author summary

Dengue fever is highly endemic in Vietnam, while scrub typhus is recognized as a re-emerging neglected disease. Both diseases are likely to account for more than half of the acute undifferentiated fever burden in Vietnam. However, scrub typhus is a bacterial disease requiring antimicrobial treatment, while dengue fever—of viral etiology—does not. Misdiagnosis and treatment delays cause potentially severe or fatal complications among scrub typhus patients, even though it is easily treatable. In this study, we used simple clinical and laboratory markers, which were identified upon admission of 221 PCR-confirmed scrub typhus cases and 387 NS1-positive dengue fever patients from Khanh Hoa province to identify the differences between scrub typhus and dengue. We found seven predictors that served to construct a simple clinical decision tree, holding great potential to distinguish scrub typhus from dengue using readily available clinical or laboratory findings. These predictors can strongly support medical staff in identifying scrub typhus cases from dengue, without using sophisticated diagnostic tests, and could improve the quality of diagnoses and appropriate treatment strategies at the primary health care level–especially in areas where scrub typhus and dengue fever are co-endemic in Vietnam and many parts of Asia and where diagnostic tests are not readily available.

## Introduction

Scrub typhus and dengue fever are major under-diagnosed causes of febrile illness in many parts of Asia [1–8]. Scrub typhus and dengue fever together account for approx. 30–40% of the leading etiologies of acute undifferentiated fever in Thailand]. Sero-epidemiological data suggest that *Orientia tsutsugamushi* infection is common across Southeast Asia, with seroprevalences ranging from 9–28% [10,11]. Case fatality rates from areas of reduced drug-susceptibility are reported at 12–14% for South India and northern Thailand, respectively [11]. High mortality rates were reported for complicated scrub typhus with central nervous system involvement (14%), multi-organ dysfunction (24%) and high pregnancy miscarriage rates with poor neonatal outcomes [12,13].

After approximately half a century of neglect, scrub typhus is beginning to receive more attention as an important cause of non-malarial febrile illness in Vietnam. Recent reports highlight scrub typhus as a disease of high clinical relevance and expanding (documentation of) distribution due to a notable recent increase in the number of diagnosed and reported cases [14,15]. In the 1960s, scrub typhus was considered a common disease among American veterans in Vietnam and an endemic disease in the midlands and mountainous forests of Vietnam, but after the discovery of Chloramphenicol the general interest in rickettsial diseases declined gradually with the availability of an effective antimicrobial [16,17]. In Vietnam only a limited number of cases were registered after the 1970s, but the increasing reports of scrub typhus in recent years suggest a re-emerging trend of this rickettsial illness with documented geographical expansion and distribution within the population of Vietnam [8,15,18]. Results from various causes-of-fever studies in Southeast Asia have confirmed the importance of this easily treatable rickettsial disease [9,15,18]. Scrub typhus is a serious disease if untreated in elderly; the median mortality is 6% if untreated, and mortality increases with age (over 50 years old mortality >45%), while case fatality risks can reach 12–13% in South India or North Thailand [11,19,20].

Dengue has made a substantial impact in Vietnam over the two past decade and is unequivocally the leading cause of febrile illness throughout the country [21–24]. Dengue has been extensively studied and its economic impact assessed; recent studies have estimated that it is responsible for 39,884 disability-adjusted life years (DALYs) annually, representing an economic burden of US$94.87 million per year (2016) [23]. Vietnam is an endemic area for dengue fever, and the level of knowledge about the disease and its management in the population was promoted through broad publicity and knowledge dissemination (mainly TV and internet) [25,26]. Dengue incidence per 100,000 population increased steadily from 32.5 in 2000, to 120.0 in 2009, and was 149.9 in 2018 in Vietnam [27–29]. The incidence distribution of dengue is higher and more consistent in the south than in the north of Vietnam.

The capacity for diagnosis (rapid and confirmatory tests) for scrub typhus in hospitals remains limited [30,31]. The standard reference assays for scrub typhus antigen are polymerase chain reaction (PCR) and serological diagnosis (ELISA), which are expensive, require expertise and sophisticated laboratory equipment. Although testing for prevalent bacterial infections informs treatment and is cost-effective, access to useful tests is scarce [32]. For dengue, the NS1 antigen or combined NS1/IgM rapid diagnostic tests are highly appropriate for the early diagnosis of dengue infection as they are readily available, easy-to-use, inexpensive, accurate and cost effective compared to dengue ELISAs and PCR assays [33–35]. However, these tests are not readily available where needed most, especially at the primary health care level or in rural, tribal areas [36].

The similarities upon presentation of these two common causes of febrile illness complicate clinical management decisions at all health care levels of the country, from the primary health care centers to even the national tertiary hospital [37]. Non-specific symptoms such as high fever, headache, skin rash or myalgia are common to both scrub typhus and dengue, but different treatment strategies are required [38–41]. Frequent misclassification of undifferentiated febrile illnesses delay the diagnosis and treatment especially for scrub typhus [8,37]. Approx. one million cases of scrub typhus occur each year, which—with an estimated 6% case fatality rate–account for a substantial mortality and economic burden for an easily-treatable disease. Improving access to diagnosis and appropriate antibiotic treatment would have an important impact [42]. At the Vietnam national referral hospital a mortality rate mortality is estimated at 1.2% among confirmed patients, but numbers in district and community health care centers remain elusive [18].

Against this background, we conducted this study to improve differentiation between scrub typhus and dengue fever using admission clinical manifestations and routine blood tests, aiming to identify simple predictors based on their probability, when no diagnostic test is available.

## Methods

### Ethical statement

Ethical approval was provided by the Scientific and Ethical Committee in Biomedical Research, Hanoi University of Public Health (No. 382/2018/YTCC-HD3 and No.329/2019/ YTCC-HD3) and by the Ethics Committee of Northwestern and Central Switzerland (Ethikkommission Nordwest- und Zentralschweiz, EKNZ) (BASEC-Nr-2018-00974). All data retrieving procedures at the five sites were approved by Provincial Health Department of Khanh Hoa; the document No 2192/ SYT-NVY was signed by the Directors of the five study hospitals (16 August 2018). For the scrub typhus study in 2019 all participants provided written informed consent prior to study enrollment and sample collection.

### Study site

Khanh Hoa province lies in the coastal South Central region of Viet Nam. With a population of 1.2 mio (2019) in its 9 districts/townships, it covers 5.2 km^2^ (2011) and includes 200 islands. Khanh Hoa has a tropical savannah climate and is a well-known tourism center in Viet Nam with over half a million visits per month; half of these are provincial residents. Nha Trang Bay in Khanh Hoa is an official member of the World’s Most Beautiful Bay Club since 2003 [43].

Khanh Hoa is hyper endemic for dengue fever, and was a major hotspot among the 11 provinces in the central Vietnam with an average of 39,876 cases/100,000 population/year during 2011–2018 [21,44]. Dengue incidence peaked at 40,204 cases /100,000 in 2016, and decreased to 920/100,000 in 2019. In the 2020 national report Khanh Hoa ranked 2^nd^ among 63 provinces in Vietnam with 295.3 cases/100,000 population [45].

Khanh Hoa is recognized as endemic for scrub typhus since WWII. Scrub typhus was first reported in Khanh Hoa in a retrospective study of United States Air Force personnel at Cam Ranh Bay in 1969 [46]. From 2008 to 2010 there were 469 cases of scrub typhus reported in the province [47]. During 2013–2014 period, the Pasteur Institute in Nha Trang confirmed 201 of 321 suspected cases of scrub typhus in the 5 hospitals in Khanh Hoa [48].

### Study design

**Fig 1:**
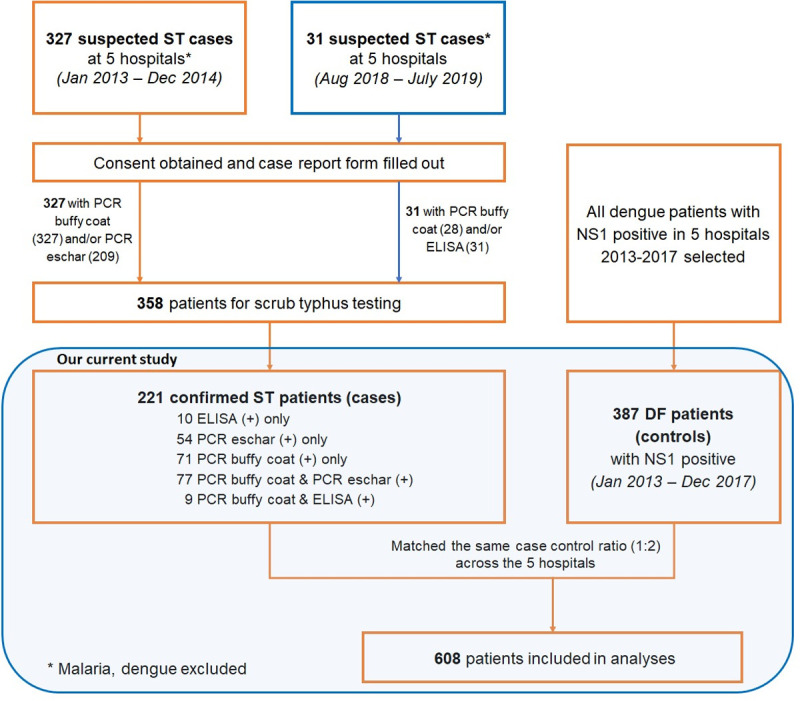
Investigational procedures and protocol for patients included in the study.

This retrospective descriptive study included 608 patients consisting of 221 and 387 confirmed acute cases of scrub typhus and dengue fever respectively. Full medical records were accessed from the 5 major hospitals of Khanh Hoa (Provincial Hospital, Ninh Hoa branch provincial hospital, Dien Khanh district hospital, 87 Army hospital, Ninh Diem Hospital). The enrollment and selection procedures are presented in Fig 1.

The data of all patients hospitalised at the five hospitals with a diagnosis of “suspected scrub typhus” (Jan 2013—Dec 2014 and Aug 2018—Jul 2019) or “dengue fever” (2013–2017) were collected. We aimed to test following hypothesis: “There is no difference in clinical manifestations and routine blood testing results between scrub typhus and dengue fever inpatients upon hospital admission”.

All diagnostic assays and clinical assessments during admission and hospitalization in the five hospitals were made by trained local laboratory staff and physicians respectively, as part of routine clinical management, and following the scrub typhus “suspected case” definitions (criteria stated below) and the well-established “dengue fever” selection criteria. From Jan 2013 to Dec 2014, after excluding malaria, dengue and other diagnoses, 327 patients fulfilled the “suspected scrub typhus” definitions on admission and were enrolled to the study; 209 eschar samples were collected, and all provided admission blood samples. From Aug 2018 to Jul 2019, 31 patients with “suspected scrub typhus” on admission were enrolled and all provided admission blood samples. In total, 358 “suspected scrub typhus” patients were enrolled, of which 221 were confirmed to be scrub typhus.

As scrub typhus is by far the more neglected disease, we included all confirmed scrub typhus patients and randomly selected two controls from the dengue fever patient group as non-scrub typhus case controls for further analysis. These confirmed cases and random controls were assigned in a 1:2 ratio across the five hospitals. All dengue patients had a documented NS1 positive test result and presented without shock symptoms (n = 378). Dengue patients with malaria co-infections (positive rapid diagnostic test (RDT) and Giemsa thin film) were excluded. Medical records were available for all dengue patients documented at the five hospitals during 2013–2017. In total, medical records of 608 scrub typhus and dengue cases were collected and included in analyses.

### Diagnostic assays

Blood specimens from all enrolled patients were taken by trained laboratory technicians and if an eschar was present, swabs of the eschar area were collected at the respective hospitals upon admission, before transfer to the Nha Trang Pasteur Institute, Vietnam, for PCR and ELISA testing [49].

### PCR assays

During 2013–2014, a quantitative SYBR green real-time PCR with primer designed from GroEL gene [50] was used to identify the presence of *O*. *tsutsugamushi* in 327 patients with 327 buffy coat samples and 209 eschar swab specimens. During 2018–2019, an in-house semi nested PCR [49], validated by the qualitative SYBR green real-time PCR [50], for detection of partial 56-Kda outer membrane protein gene was used to identify the presence of *O*. *tsutsugamushi* in 28 patients with 28 PBMCs samples (3 patients did not provide buffy coat samples). Primers used for the semi-nested PCR includes 2 forwards primers with the sequence of (F1): CAATGTCTGCRTTGTCRTTG; (F2): CCKTTTTCIGCTRGTGCGATAG and 1 reverse primer with sequence of (R): ATAGYAGGYTGAGGHGGYGTAAG. In total, there were 564 specimens available, from 358 suspected scrub typhus cases, including 355 buffy coat samples and 209 eschar swabs for PCR testing.

### ELISA assays

The Scrub Typhus Detect IgM ELISA (part no. 500242, Lot no. XM5033; InBios International Inc., Seattle, WA, USA) was used for IgM detection all 31 serum samples of patients enrolled from 2018–2019. This ELISA uses recombinant p56kD type specific antigens of *Orientia tsutsugamushi* Karp, Kato, Gilliam, and TA716 strains. The manufacturer’s methods were followed exactly. All sera were tested at a 1:100 dilution and absorbance was determined at 450 nm (OD@450 nm) using a microplate reader to give a final optical density (OD) result. The OD cut-off applied was 1.00 with a sensitivity of 91.5% and specificity of 90.9% for admission samples to confirm cases among suspected scrub typhus infection, as reported previously [51,52].

### Case definitions and selection criteria

“Suspected scrub typhus”: Age not restricted with a febrile illness (axillary temperature ≥37.5°C) and at least one of the following criteria needed to be fulfilled:

- Presence of an eschar
- Suspected dengue fever with a negative dengue NS1 test result
- Suspected malaria with a negative malaria test result (microscopy, RDT)
- Persisting or undifferentiated fever (≥10 days fever)

From August 2018 to July 2019, the same criteria were re-phrased to reflect more detail:

- Age ≥ 16 years old
- Patient with acute fever (axillary temperature ≥37.5°C) and having had at least one of the following twelve secondary findings: eschar, nonspecific skin rash, headache, myalgia, retro-orbital pain, congestion of the conjunctival blood vessels, tinnitus, lymphadenopathy (regional/body), hepatomegaly, splenomegaly, dry cough, dyspnoea without upper respiratory tract discharge.
- Exclusion criteria: Patients diagnosed with malaria, dengue fever (confirmed by NS1), measles, influenza, bacterial pneumonia, urinary tract infections.

Confirmed acute cases

- Scrub typhus: Patients with a positive PCR result (buffy coat or eschar swab specimens) or positive IgM ELISA result (optical density [OD] of ≥ 1.0) for *O*.*tsutsugamushi* by the Institute Pasteur Nha Trang reference laboratory, in Vietnam.
- Dengue fever: Patients with a positive dengue NS1 antigen test (NS1) performed on site at each of the 5 hospitals. Patients with clinical symptoms of shock were excluded (due to the specific symptoms associated; *i*.*e*. circulatory failure, pronounced tachycardia with weak and narrow pulse pressure, hypotension, cold, clammy skin, abnormal mental status, oliguria, metabolic acidosis, restlessness; or profound shock with undetectable blood pressure or pulse [53])
- Co-infections in scrub typhus and dengue fever cases with a positive malaria RDT and/or Giemsa staining method were excluded from the study.

### Sample size considerations

A Monte Carlo simulation [54] showed that, 200 scrub typhus cases and 400 dengue fever controls would be sufficient to keep the estimation error of the area under the curve (AUC) associated with the prediction of scrub typhus within about 3.5% of the true value with 95% certainty, provided that the true value of AUC is ≥ 80% [55]. The precision increases with increasing AUC. The AUC was used because it represents overall performance of a prediction score. It allows to find a good threshold for the prediction score to distinguish between patients with and without the specific disease. The same ratio between cases and controls was applied across the five hospitals to avoid potential confounding by differences in the diagnosis capacities of physicians across hospitals.

### Data sources and data quality assurance

Complete medical records of all 608 scrub typhus (cases) and dengue patients (controls) were retrieved from the paper-based medical record filing cabinets stored at the storing units of the five hospitals in Khanh Hoa. The cases and controls (1:2 ratio) were associated with the same hospital, and the following data was extracted from the patients’ medical record: clinical manifestations, routine blood testing results, method of diagnosis and management upon admission.

A structured data collection form was used to retrieve medical records; All skips, data format requirements, cross check, and data constraints were designed for quality assurance, prior to building the form on Open Data Kit (ODK) [56], before uploading to the web-based server http://sg.smap.com.au/, from where it was downloaded onto Android devices (Samsung tablets). The Open Data Kit community produces free and open-source software for collecting, managing, and using data in resource-constrained environments [56]. The use of mobile data capture technology such as ODK and Android mobile devices have proven their efficiency and cost-effectiveness in cross-sectional surveys [57,58] and are recommended by the WHO [59].

Four trained data collectors with experience in scrub typhus studies from the Department of Epidemiology, Institute Pasteur, Nha Trang used this e-form programmed on Samsung tablets to collect data. At the end of each day, the data supervisor checked the total numbers of forms and randomly 15% of the forms collected by each data collector, and any incomplete forms were completed. The ODK program checked for missing data, so that the form could only be closed and marked as “finished” when all information was provided (no information = 99999). All completed forms were uploaded to the web-based server at http://sg.smap.com.au/ at the end of each working day. Copies were stored in the tablets, and all collected data was secured in the web-based server, and downloaded for subsequent analyses in STATA.

### Statistical analyses

Identical variables were recorded for cases and controls, with the dependent variable chosen as scrub typhus (Yes/No). Primary independent variables were: fever, days of fever on admission, headache, hemorrhage, hepatomegaly, splenomegaly, lymphadenopathy (regional/body) and the basic blood laboratory results. A training data set was built by randomly selecting 60% of cases and 60% of controls. The remaining data was used for validation of the prediction models.

Descriptive statistics included counts, proportions and percentages for qualitative variables, and medians and interquartile ranges (IQR) for quantitative variables. Comparisons of demographic, social, and laboratory variables between patients and control groups were conducted using the Fisher’s exact test and the Mann-Whitney U test, as indicated. Logistic Regression (LR) was applied to derive a prediction model for the dichotomous dependent variable (presence vs. absence of scrub typhus).

First, potential predictor variables for scrub typhus other than eschar were considered one by one in the training data set. The Bayes information criterion (BIC) was applied to determine the variables to be considered in the initial multivariable model. This initial model was then reduced using backward selection based on the BIC. A variable remained in the model if its removal increased BIC. The optimal cut points for the predicted probabilities of a patient having scrub typhus as opposed to dengue fever were determined by maximizing the sum of sensitivity and specificity (i.e., the index of Youden). We derived 3 models, i.e. a model without laboratory variables, one only including laboratory variables, and one with both clinical and laboratory variables (no using eschar variable) (Table 1). In a further step, the variable eschar, which perfectly predicts scrub typhus, was added to the prediction model by setting the predicted probability of scrub typhus to one among patients with eschar. The resulting model was then applied to the validation data set and the receiver operating characteristic curves (ROC-curves) were generated to compare the performance of the model in both data sets based on the area under the curve (AUC). Finally, the model was fitted in the entire data set. This could be justified by the good performance of the training model in the validation data set.

**Table 1:** Demographic, clinical, diagnostic and laboratory characteristics of patients at admission.

	Scrub typhus	Dengue fever	OR (95%CI)	P-value^a^
**Demographics and History**				
Male, n (%)	124/221 (56.1%)	204/387 (52.7%)	0.87 (0.63–1.22)	0.419
Age, median (IQR)	33 (22–45)	20 (10–31)	1.03 (1.02–1.04)	**<0.001**
Main occupation in nature, n (%)*	85/221 (38.5%)	58/387 (15.0%)	3.55 (2.40–5.23)	**<0.001**
Referral, n (%)	26/221 (11.8%)	37/ 357 (10.4%)	1.15 (0.68–1.96)	0.600
Days of fever on admission (> = 37.5°C), median (IQR)**	5 (3–7)	3 (2–4)	1.68 (1.52–1.85)	**<0.001**
**Clinical presentation at admission** ^**#**^				
Symptoms				
Headache, n (%)	145/220 (65.9%)	236/387 (61.0%)	1.24 (0.88–1.75)	0.228
Myalgia, n (%)	92/221 (41.6%)	118/387 (30.5%)	1.63 (1.15–2.29)	**0.006**
Retro-orbital pain, n (%)	19/220 (8.66%)	8/387 (2.07%)	4.48 (1.93–10.4)	**<0.001**
Rigors/chills, n (%)	29/220 (13.2%)	4/387 (1.0%)	14.5 (5.04–42.0)	**<0.001**
Dry cough, n (%)	36/220 (16.4%)	41/387 (10.6%)	1.65 (1.02–2.67)	**0.041**
Abdominal pain, n (%)	28/219 (12.8%)	60/387 (15.5%)	0.80 (0.49–1.29)	0.362
Diarrhea (at least 3 days), n (%)	10/220 (4.55%)	6/387 (1.55%)	3.02 (1.08–8.44)	0.035
**Physical signs**				
Body temperature > = 38° C, n (%)	155/216 (71.8%)	224/315 (71.1%)	1.03 (0.70–1.51)	0.871
Heart rate > 90/min, n (%)	112/221 (50.7%)	223/387 (57.6%)	0.76 (0.54–1.05)	0.098
Respiratory rate > 22/min, n (%)	21/203 (10.3%)	83/386 (21.5%)	0.42 (0.25–0.70)	**0.001**
Hypotension, n (%)	32/218 (14.7%)	54/348 (15.5%)	0.94 (0.58–1.51)	0.787
Eschar, n (%)	198/221 (89.6%)	0/387 (0.00%)	1.00 (1.00–1.00)	.
Rash, n (%)	13/221 (5.88%)	25/387 (6.46%)	0.88 (0.67–1.16)	0.777
Hemorrhagic signs (Petechial hemorrhage (epistaxis, bleeding gums, organs), skin hemorrhage, n (%)	10/221 (4.52%)	97/387 (25.1%)	0.14 (0.07–0.28)	**<0.001**
Regional lymphadenopathy (>1cm), n (%)	75/221 (33.9%)	2/387 (0.52%)	98.9 (24.0–408)	**<0.001**
Hepatomegaly and/or splenomegaly, n (%)	3/221 (1.36%)	5/387 (1.29%)	1.17 (0.19–7.05)	0.946
Pharyngo-laryngitis, n (%)	36/220 (16.4%)	41/387 (10.6%)	0.33 (0.17–0.65)	**0.041**
Documented dyspnoea, n(%)	8/220 (3.64%)	3/387 (0.78%)	4.83 (1.27–18.4)	**0.021**
Lung crepitation, n (%)	9/220 (4.09%)	2/386 (0.52%)	8.19 (1.75–38.3)	**0.007**
Fatigue, n (%)	90/221 (40.7%)	171/387 (44.2%)	0.87 (0.62–1.21)	0.407
Malaise, n (%)	2/219 (0.91%)	3/387 (0.78%)	1.18 (0.20–7.12)	0.857
Nausea, n (%)	15/219 (6.85%)	50/387 (12.9%)	0.50 (0.27–0.91)	**0.022**
Vomiting, n (%)	12/221 (5.43%)	35/387 (9.04%)	0.58 (0.29–1.14)	0.112
Lung crepitation and/or documented dyspnoea, n (%)	14/220 (6.36%)	4/386 (1.04%)	6.49 (2.11–20.0)	**0.001**
Gastrointestinal findings, n (%)	44/218 (20.2%)	109/387 (28.2%)	0.64 (0.43–0.96)	**0.031**
Clinical severity, n (%)	73/200 (36.5%)	122/347 (35.2%)	1.06 (0.74–1.52)	0.752
**Laboratory findings** ^**##**^				
WBC (10^3^/mm^3^), median (IQR)	7.2 (5.1–9.9)	4.1 (3.1–6.4)	1.35 (1.27–1.44)	**<0.001**
NEU (10^3^/mm^3^), median (IQR)	4.5 (3.1–5.9)	2.5 (1.5–4.2)	1.36 (1.25–1.47)	**<0.001**
Lymphocytes (10^3^/mm^3^), median (IQR)	1.7 (1.1–2.7)	0.9 (0.6–1.3)	1.95 (1.65–2.30)	**<0.001**
N/L Ratio (neutrophils/lymphocytes)	2.5 (1.6–3.8)	2.8 (1.4–5.0)	0.89 (0.83–0.95)	**<0.001**
HCT %, median (IQR)	38 (34.7–42.0)	37.7 (35.0–40.8)	1.00 (0.97–1.04)	0.827
RBC (10^12^/L), median (IQR)	4.5 (4.2–4.8)	4.5 (4.2–4.8)	0.89 (0.69–1.15)	0.377
PLT (~G/L), median (IQR)	121 (91–160)	127 (86–177)	1.00 (1.00–1.00)	0.757
HGB (g/dL), median (IQR)	12.5 (11.3–13.5)	12.4 (11.6–13.5)	0.96 (0.86–1.07)	0.497
Creatinine (umol/L), median (IQR)	84 (74–102)	81 (70–98)	1.00 (0.99–1.01)	0.975
AST (U/L), median (IQR)	97.5 (69–172)	81 (42.0–118)	1.01 (1.00–1.01)	**0.001**
ALT (U/L), median (IQR)	108 (58–166)	62 (30.5–99.5)	1.01 (1.00–1.01)	**0.001**
AST and/or ALT ≥45 U/L (n, %)	68/148 (45.9)	8/32 (25.0)	2.55 (1.08–6.04)	**0.034**

^a^ Significant predictor variable on univariate logistic regression analysis (p<0.05) are indicated in bold.

* An occupation in nature: farmer, fisherman, working in forest.

**Fever: tympanic temperature >37.5°C measured by axillary method

# Clinical presentation

Gastrointestinal findings: at least one of abdominal pain, vomiting, nausea, jaundice, hepatomegaly, splenomegaly

Clinical severity–at least one of these: intubation; respiratory rate >30/min; pulse >100/min; systolic blood pressure <90mmHg or >160mmHg, or diastolic blood pressure <60mmHg;

## Laboratory reference range: WBC 4–10 G/L, NEU 2.6–7.0 G/L, L 1.2–3.8 G/L, HCT: 0.33–0.50L/L, PLT 150–450 G/L, HGB 12.0–16.5 g/dL (International Standard unit)

An alternative approach for discriminating between scrub typhus and dengue fever consisted in deriving binary decision trees using the CART (classification and regression trees) method [9,60–63]. The trees were developed for the entire dataset and were pruned based on the inbuilt cross-validation statistic of the CART program [64]. Each node of the tree represents a binary decision and the leaves of the tree are assigned to the diagnosis of either scrub typhus or dengue fever. Trees were pruned in order to avoid overfitting of the data. As for the regression-based prediction models, the model performance was assessed based on the sensitivity and specificity of predictions and on the index of Youden (i.e., the sum of sensitivity and specificity minus 1). The probability cut points used to assign final leaves to scrub typhus or dengue fever were chosen such as to maximise the index of Youden.

Descriptive and logistic regression analyses were conducted using STATA software version 14, while CART-analyses were conducted using R-software (Version 1.1.456–2009–2018 RStudio, Inc.)

## Results

### Socio-demographic and epidemiological findings

We included all 221 cases of scrub typhus and 387 cases of dengue in the analyses, reflecting approximately a 1:1,75 assignment. There were significant differences in age, occupation and number of days with fever before admission. The median (interquartile range—IQR) ages of the scrub typhus and dengue patients were 33 (22–45) and 20 (10–31) years, respectively (p<0.001). The proportion of occupation in nature was higher among patients with scrub typhus (38.5%) than among patients with dengue fever (15.0%) (p<0.001). There was also a significant difference in the days of fever on admission, between the patients with scrub typhus (median = 5 days, IQR = 3–7 days) and those with dengue fever (median = 3 days, IQR = 2–4 days) (p<0.001).

**Fig 2:**
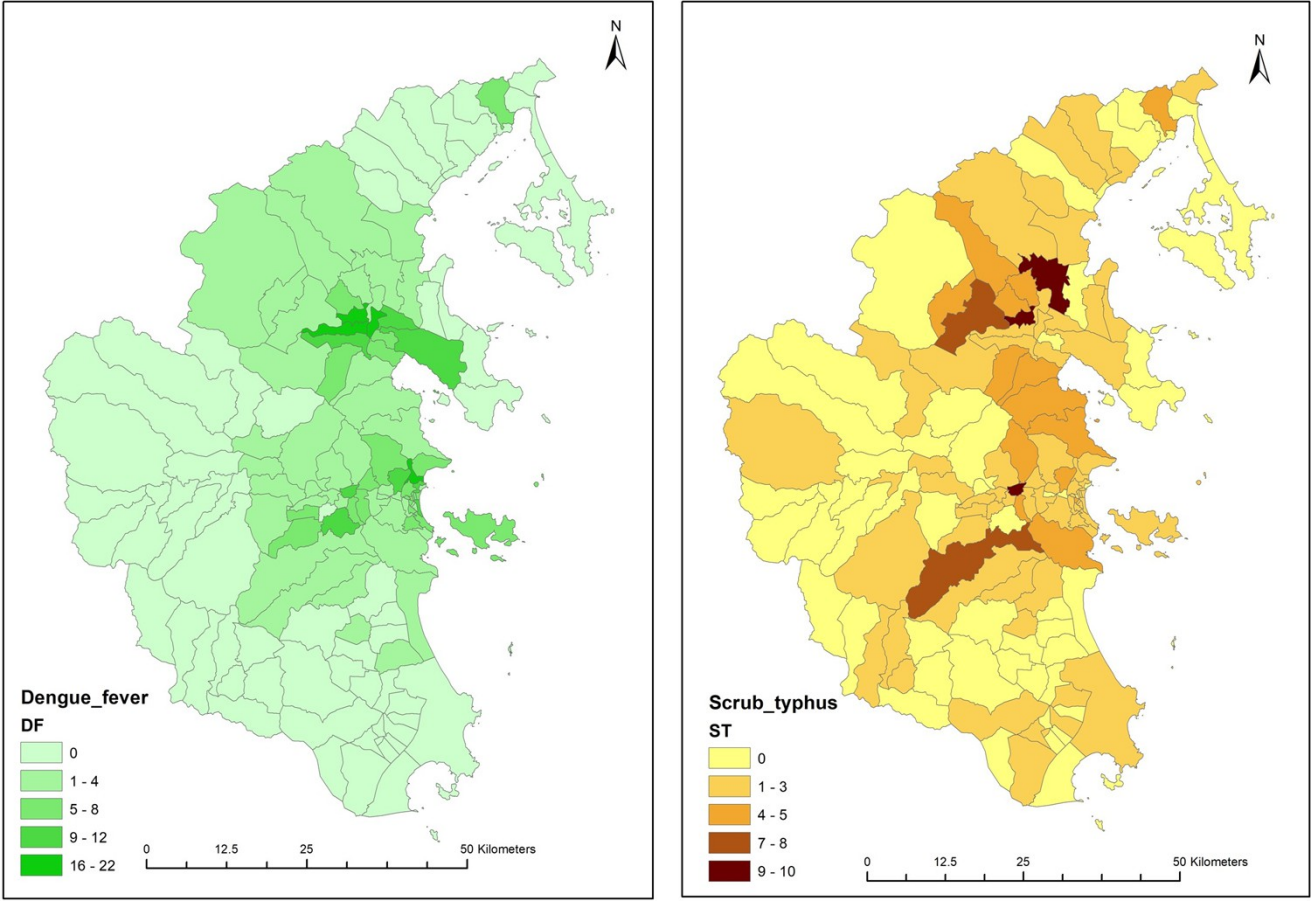
The geographic distribution in Khanh Hoa of all scrub typhus (n = 221) and dengue fever (n = 387) confirmed cases in this study is depicted in these maps. (note: this figure was created using ArcGIS® software by Esri (www.esri.com). Source of the administrative layer of Vietnam was obtained from the website (http://www.diva-gis.org/datadown#google_vignette) which is free for community users).

The geographic distribution of the scrub typhus and dengue fever confirmed cases in this study is demonstrated in Fig 2. Scrub typhus cases occurred in all 8 districts in Khanh Hoa, and a similar distribution of dengue and scrub typhus confirmed cases was seen across the communes.

### Clinical and laboratory findings

Clinical manifestations and laboratory findings are summarised in Table 1. The strength of diagnostic factors for scrub typhus as opposed to dengue fever is expressed by the respective odds ratio. In the two scrub typhus cohorts, an eschar prevalence of 93.1% was seen among the 202 scrub typhus cases seen from 2013–2014, and 52.6% among the 19 scrub typhus cases seen in 2018–2019. The presence of an eschar is a vital diagnostic clue for scrub typhus; if the physician finds an eschar, it informs diagnosis and treatment of scrub typhus patients. But if there is no eschar, often scrub typhus is missed, and other differential diagnoses considered. Therefore, we conducted the analyses in datasets with and without the eschar variable for more feasible prediction approaches.

Patients with scrub typhus were more likely to have regional lymphadenopathy (>1cm), with a high odds ratio (OR) of 98.9; 95% confidence interval (CI): 24.0–408. This was followed by rigors/chills OR = 14.5 (95% CI: 5.04–42.0); lung crepitation OR = 8.19 (95% CI: 1.75–38.3); documented dyspnoea OR = 4.83 (95% CI: 1.27–18.4); retro-orbital pain OR = 4.48 (95%CI:1.93–10.4); diarrhea (at least 3 days) OR = 3.02 (95%CI:1.08–8.44) and myalgia OR = 1.63 (95%CI:1.15–2.29). On the other hand, patients with scrub typhus were significantly less likely to have pharyngo-laryngitis OR = 0.33 (95%CI: 0.17–0.65); respiratory rate >22 OR = 0.42 (95%CI: 0.25–0.70); and hemorrhagic signs OR = 0.14 (95%CI: 0.07–0.28), compared to dengue fever patients.

Significant hematological blood laboratory predictors for scrub typhus included higher white blood cell count OR = 1.35 per unit increase in white blood cell (WBC) (95%CI 1.27–1.44, p ≤ 0.001); neutrophil (NEU) count, OR = 1.36 per unit increase in NEU (95%CI 1.25–1.47, p ≤ 0.001); or lymphocyte count, OR = 1.95 per unit increase (95%CI 1.65–12.3, p ≤ 0.001), and aspartate aminotransferase (AST/GOT) or alanine aminotransferase (ALT/GPT) levels ≥45 U/L with an OR = 2.55 (95%CI:1.08–6.04, p ≤ 0.034).

The significant predictors for scrub typhus in the training data set of the multivariable logistic regression model after backward selection are presented in Table 2. Besides eschar, we identified four significant clinical variables for scrub typhus and two significant routine hematological blood laboratory parameters. In the model combining these variables, the odds of having scrub typhus as opposed to dengue fever were positively associated with regional lymphadenopathy aOR = 78.2 (95%CI: 9.20–665, p <0.001); followed by an occupation in nature, aOR = 3.87 (95%CI: 1.89–7.91, p <0.001); with age over 40, aOR = 3.94 (95%CI:1.94–8.01, p <0.001); with increased days of fever on admission aOR = 1.45 per additional day (95%CI: 1.22–1.66, p <0.001); and with neutrophil count, aOR = 1.89 per unit increase (95%CI:1.54–2.32, p <0.001). On the other hand, the association with ratio of neutrophils/lymphocytes was negative with an aOR = 0.68 per unit increase (95%CI: 0.57–0.81, p <0.001).

**Table 2:** Results from multivariate logistic regression with the most relevant predictors for the presence of scrub typhus (training part)^#^.

	Clinical manifestations			Routine hematological blood laboratory			Clinical manifestations & Routine hematological blood laboratory		
	**aOR**	**95%CI OR**	**P-value**	**aOR**	**95%CI OR**	**P-value**	**aOR**	**95%CI OR**	**P-value**
Eschar (no using)									
Regional lymphadenopathy	96.3	12.2–759	**<0.001**				78.2	9.20–665	**<0.001**
An occupation in nature	3.75	2.02–6.96	**<0.001**				3.87	1.89–7.91	**<0.001**
Age over 40	3.39	1.78–6.46	**<0.001**				3.94	1.94–8.01	**<0.001**
Days of fever on admission (Nr)	1.49	1.30–1.71	**<0.001**				1.42	1.22–1.66	**<0.001**
Neutrophil count				2.09	1.75–2.50	**<0.001**	1.89	1.54–2.32	**<0.001**
Ratio of N/L (neutro/lymph)				0.61	0.53–0.71	**<0.001**	0.68	0.57–0.81	**<0.001**
	**AUC**	**95% CI**		**AUC**	**95% CI**		**AUC**	**95%CI**	
ROC–analysis (n = 364)	0.862	0.823–0.896		0.831	0.790–0.869		0.912	0.878–0.939	

^#^ Results from multivariate logistic regression with the most important predictor variables in the training data set (n = 364). Initial clinical manifestation variables considered included days of fever on admission, myalgia, retro-orbital pain, rigor, hemorrhagic signs (epistaxis, bleeding gums, organs, or skin hemorrhage), regional lymphadenopathy (>1cm); at least one of: lung rales or documented dyspnoea, pharyngo-laryngitis, respiratory rate <22/min; an occupation in nature, 5-year age groups). The initial routine hematological blood laboratory variables included neutrophil count, lymphocyte count, ratio (Neutrophils/Lymphocytes), AST (GOT), ALT (GPT). The present models was obtained by backward selection guided by the Bayes information criterion (BIC).

### ROC curves

Besides eschar, we found the most relevant predictors of scrub typhus to be: increased days of fever on admission, regional lymphadenopathy, neutrophil count, ratio of N/L (neutrophils/lymphocytes), age over 40, and an occupation in nature (Table 2). ROC curves were generated to visualise the performance of these seven variables in differentiating between scrub typhus and dengue fever, using mulvariable logistic regression (M-LR).

**Fig 3:**
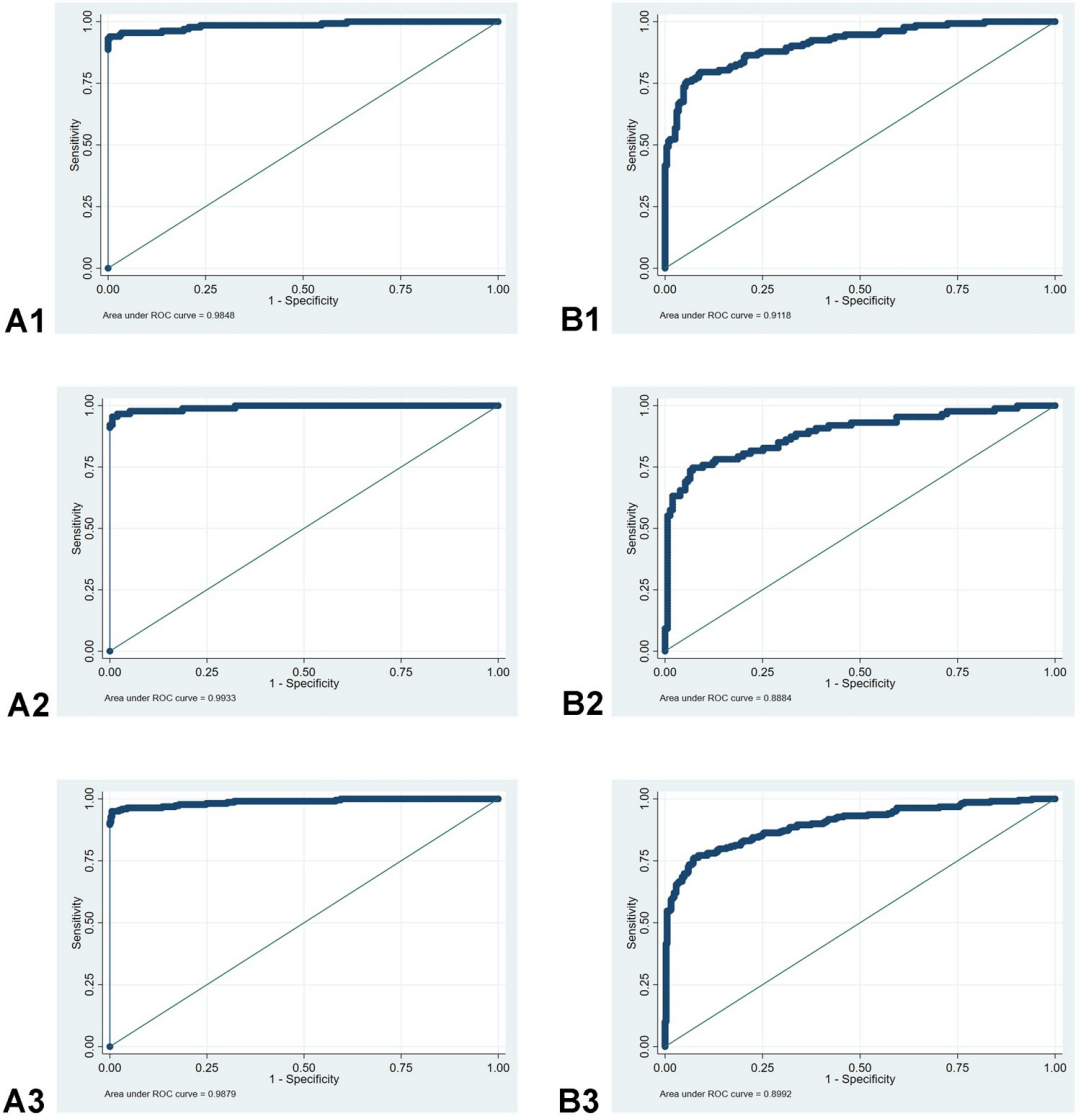
ROC curves-performance of prediction models for scrub typhus as opposed to dengue fever, using M-LR. Panels A1, A2, A3: the variable “eschar” was added into the prediction model; panels B1, B2, B3: “eschar” was not added into the prediction model.

When adding eschar into the prediction model, i.e., by setting the probability of scrub typhus to 1 in patients with eschar, the areas under the ROC curve increased to 0.985 (95% CI:0.964–0.994), 0.993 (95% CI: 0.971–0.999) and 0.988 (95% CI: 0.976–0.995) in the training data set, validation data set and the whole data set, respectively (Fig 3A1 and 3A2 and 3A3, respectively). When not using the eschar variable, the areas under the ROC curve for the 6 remaining variables were 0.912 (95% CI: 0.878–0.939), 0.888 (95% CI: 0.842–0.925) and 0.899 (95% CI: 0.873–0.922) in the training data set, the validation data set and the entire data set, respectively (Fig 3B1 and 3B2 and 3B3, respectively).

### Decision tree analysis

**Fig 4:**
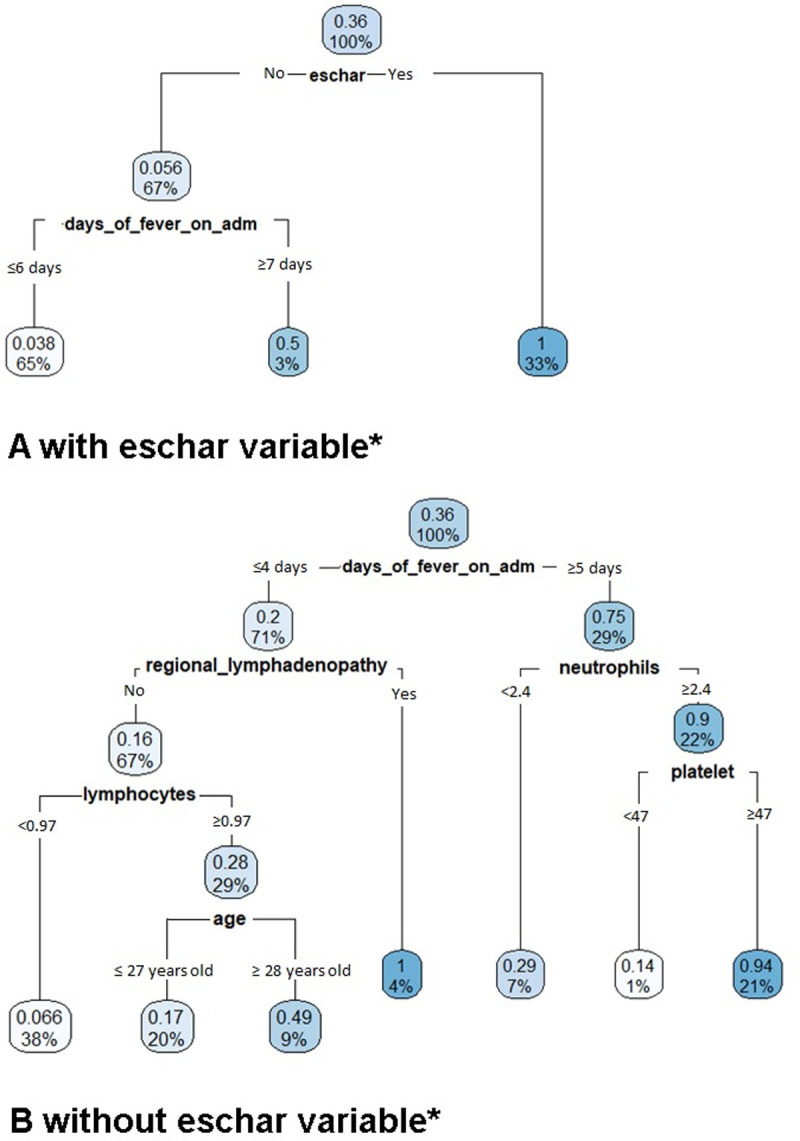
Regression tree for scrub typhus using the entire data set. * Panel A: tree obtained when offering the variable “eschar”; panel B: tree obtained when not offering the variable “eschar”.

In a second approach, CART analysis was applied to derive binary decision trees for distinguishing scrub typhus from dengue fever in the full data set (Fig 4). Each node of the tree represents a binary decision and the leaves of the tree are assigned to the diagnosis of either scrub typhus or dengue fever. In each node, 2 numbers are presented at the decision node level. The upper number indicates the positive predictive value associated with the respective node. The higher the probability of patients being scrub typhus, the darker the color of the node. The bottom number shows the percentage of patients at the respective node.

If the eschar variable was offered, the resulting tree involved the two variables “eschar” and “days with fever at admission” (Fig 4A). At the second decision node level, with “eschar” being positive, the probability of being scrub typhus is 1 (100%), and this node accounts for 33% of all patients. Among patients without “eschar” those with seven or more days of fever on admission had a probability of 50% of being diagnosed with scrub typhus. They accounted for 3% of the total sample.

When not offering “eschar” variable, the six variables days of fever on admission, regional lymphadenopathy, lymphocyte count, neutrophil count (without regional lymphadenopathy), platelet count and “age over 28 years old” were selected by the algorithm (Fig 4B). In detail, patients with ≥5 days of fever on admission had a probability of 75% of being scrub typhus–in a cohort of scrub typhus and dengue fever patients. This criterion was satisfied by 29% of the patients. Moreover, the positive predictive value of scrub typhus increased to 90% for patients who additionally had a neutrophil count ≥ 2.4 (x10^3^/mm^3^), and it further increased to 94% if patients additionally had a platelet count ≥ 47 (G/L).

Thus, we found slight differences between the two alternative approaches: while the CART tree included “platelet count”, the regression approach resulted in the inclusion of “an occupation in nature”. Moreover, the “age” cut point in the tree is at 28 years while we had found a cut-off of 40 years to discriminate well when deriving the regression model.

Table 3 presents the most relevant predictors of scrub typhus including eschar, regional lymphadenopathy, an occupation in nature, age, increased days of fever on admission, increased neutrophil count, decreased neutrophil/lymphocyte ratio, and platelet count ≥ 47 G/L, revealed by CART (using R) and by the multivariate logistic regression (M-LR) approach (using STATA).

**Table 3:** The most relevant predictors of scrub typhus selected by CART (using R) and by the multivariate logistic regression (M-LR) approach (using STATA).

STRONG PREDICTORS OF SCRUB TYPHUS
1. Eschar
2. Regional lymphadenopathy
3. An occupation in nature*
4. Higher age**
5. Increased days of fever on admission (Nr)
6. Increased neutrophil count
7. Decreased Ratio (Neutrophils/Lymphocytes)▲
8. Platelet count ≥ 47 G/L ^#^

* Fishing/agriculture/working in forest, only in M-LR

** Age over 40 in M-LR and age over 28 in CART

▲ Ratio (neutro/lymph) in M-LR and Lymphocytes in CART

^#^ Only in CART

### Model validation

The results from the M-LR model, using the set of 7 predictors: eschar, increased days of fever on admission, regional lymphadenopathy, an occupation in nature, increased neutrophil count, decreased ratio of N/L (neutrophils/lymphocytes), age over 40 was very sensitive and very specific for defining scrub typhus (using whole data, sensitivity = 93.7%, specificity = 99.5%, Youden = 0.932), when directly comparing scrub typhus and dengue fever groups. The respective values were very similar in the training and the validation data set.

The regression tree generated for scrub typhus with the two predictors eschar and days of fever on admission had a slightly lower index of Youden (0.919) and a lower specificity (96.9%), while the sensitivity was slightly higher (95%) (Table 4).

**Table 4:** Model validation: Accuracy of Scrub typhus Prediction Models derived by Multivariate Logistic Regression vs. CART.

Variables	Multivariate LR	Multivariate LR	Multivariate LR*	CART^*#*^
N	N = 364	N = 244	N = 608	N = 608
Dataset	Training	Validation	Whole data	Whole data
**Using data with eschar variable**				
Sensitivity	93.2%	94.4%	93.7%	95.0%
Specificity	99.6%	99.4%	99.5%	96.9%
Positive Predictive Value (PPV)	99.2%	98.8%	99.0%	94.6%
Negative Predictive Value (NPV)	96.3%	96.9%	96.5%	97.2%
Youden	0.928	0.937	0.932	0.919
**Using data without eschar variable**				
Sensitivity	77.3%	74.7%	76.3%	77.4%
Specificity	92.7%	91.6%	92.3%	90.7%
PPV	85.7%	83.3%	84.8%	82.6%
NPV	87.8%	86.6%	87.3%	87.5%
Youden	0.700	0.663	0.686	0.681

* after refitting the prediction model in the whole data set with the six variables: days of fever on admission, regional lymphadenopathy, an occupation in nature, neutrophil count, ratio (neutrophils/lymphocytes), and age over 40. For the derivation of the predicted probabilities using logistic regression, the model without the eschar variable was used as a basis, with the probability of scrub typhus then being changed to 1 among patients with an eschar.

^#^ Regression tree using R in entire dataset after pruning (Fig 4B).

### Using data without “eschar” variable

The binary predictor derived from the M-LR model using the six variables increased days of fever on admission, regional lymphadenopathy, an occupation in nature, increased neutrophil count, decreased ratio of N/L (neutrophils/lymphocytes), and age over 40 had a moderate sensitivity (76.3%) but a high specificity (92.3%), providing an index of Youden of 0.686. The area under the ROC-curve defined by the underlying numerical prediction score was 0.899 (95% CI: 0.873–0.922). Again, the respective statistics were very similar in the training and the validation data set.

The decision tree algorithm in the entire dataset revealed six predictors: days of fever on admission, regional lymphadenopathy, neutrophil count, lymphocyte count, platelet count, age over 28. In the CART, the tree included platelet count and age over 28. In the full data set, the index of Youden of the decision tree model (0.681) was almost identical to the one of the regression-based model (Table 4).

### Relevant findings from the models

**1. The model of demographic characteristics, epidemiological information, clinical variables to predict scrub typhus**

In the clinical model derived by M-LR without using the “eschar” variable, the most relevant clinical manifestation factors to predict scrub typhus were regional lymphadenopathy, days of fever on admission, an occupation in nature and age over 40. The clinical model worked well, and with these 4 factors, the area under ROC curve was 0.862 (95%CI: 0.823–0.896) in the training data without eschar variable (Table 2).

**2. The model of the routine hematological blood laboratory variables to predict scrub typhus**

In the M-LR model involving laboratory variables only, the most relevant routine complete blood count values to predict scrub typhus were neutrophil count and ratio of N/L (Neutrophils/Lymphocytes). The laboratory model had a higher predictive performance than the clinical model. The model with these 2 factors had an area under ROC curve of 0.831 (95%CI: 0.790–0.869) in the training data set without without eschar variable (Table 2).

**3. The model combining demographic characteristics, epidemiological information, clinical and laboratory variables to predict scrub typhus**

Combining the demographic characteristics, epidemiological information, clinical and laboratory variables, using M-LR, the seven most significant predictors for scrub typhus were; eschar, regional lymphadenopathy, days of fever on admission, an occupation in nature, increased neutrophil count, decreased ratio of N/L (Neutrophils/Lymphocytes), and age >40 years. The model with inclusion of all mentioned variables worked better than the models including the clinical or routine hematological blood laboratory variables only. With all of these factors, the area under ROC curve reached 0.988 (95%CI: 0.976–0.995) in the whole data set when including the eschar variable (Fig 3A3), and 0.899 (95% CI: 0.873–0.922) when excluding it (Fig 3B3).

The decision tree algorithm revealed the following seven most important predictors; eschar, regional lymphadenopathy, ≥5 days of fever on admission, increased neutrophil count, increased lymphocyte count, platelet count ≥ 47 G/L, and age >28 years. The tree demonstrated almost the same accuracy with the multivariate logistic regression analyses (index of Youden: 0.681 vs. 0.686), when not using the “eschar” variable (Table 4).

## Discussion

Dengue fever is highly endemic in Vietnam, but scrub typhus—although recognized as an endemic disease—remains underappreciated. Scrub typhus is probably the most prevalent under-recognized treatable cause of undifferentiated febrile illness in Vietnam [8,18,37,65]. One of the few clinical studies conducted in the national hospital in northern Vietnam suggested that up to 40.9% (273/579) of acute undifferentiated fever (AUF) patients had scrub typhus, after excluding patients with malaria, dengue fever, and typhoid fever, although this is likely an over estimation due to serological diagnostics and selection criteria of AUF patients considered as suspected rickettsial infections [8]. Dengue was responsible for one third (234/2108; 33.6%) of all acute undifferentiated fevers at the primary health care level in a year [36]. Hence, scrub typhus and dengue together are likely to contribute to more than half of undifferentiated febrile illnesses either at national referral hospital or at primary health care centers. In this study we identified simple predictors to assist in differentiating scrub typhus from dengue fever using basic clinical and laboratory parameters in Vietnam, to improve the quality of diagnoses and appropriate treatment strategies at primary health care level.

Following considerations regarding these two acute fevers need to be taken into account in Vietnam; Firstly, medical staff are not well aware of the potential causes of AUF [15,36,66], largely because robust “causes of fever” studies remain limited. Secondly, there is a strong general awareness of dengue due to the high case numbers and its impact between 2011 and 2018, the involvement of media campaigns, and with the broad availability of accurate RDTs leading almost to a perception bias towards dengue for any febrile illness in Vietnam [36]. Thirdly, the diagnostic capacity for scrub typhus (point-of-care and confirmatory assays) remains difficult and limited, even at the national referral hospital level [37]. PCR and serology are expensive, require considerable expertise and sophisticated laboratory equipment and simple RDTs are lacking [31]. Until this is improved, there is an absolute need for better predictors to inform empirical treatment or management for doctors.

### Predictors to distinguish scrub typhus from dengue fever

In a large collection of characterized patients, this study identified predictors for scrub typhus to be; i) the eschar; ii) regional lymphadenopathy; iii) an occupation in nature; iv) ≥5 days of fever on admission; v) increased neutrophil count; vi) low ratio of neutrophils/lymphocytes; vii) platelet count ≥ 47 G/L; and viii) higher age (Table 3). In a Vietnamese cohort of dengue and scrub typhus patients, these predictors can identify scrub typhus with sensitivity of 93.7%, specificity of 99.5%, with a diagnosis accuracy (ROC curve) of 0.988 (95% CI: 0.976–0.995), if an eschar is present in scrub typhus cases. In the case of no eschar, the sensitivity and specificity of this approach drops to 76.3% and of 92.3% respectively, with a diagnosis accuracy (ROC curve) of 0.888 (95% CI: 0.878–0.939). This means that applying these predictors without using any diagnostic test, would strongly support medical staff in identifying scrub typhus cases (up to 99% if an eschar is found, and 89% if not). This also highlights the importance of a thorough clinical examination, especially as eschars are often hidden in skin folds or the genital areas [67].

### Diagnostic considerations for the role of eschars

An underappreciated problem regarding the presence of eschars as a vital diagnostic clue is that their occurrence can vary broadly across different regions, and that pre-existing immunity can suppress eschar formation at the mite bite inoculation site [16,68]. Several studies in Vietnam revealed eschar prevalence across communities from 18.2% to 46.6% [8,37], while reports from other areas in Asia suggest eschar prevalence from 7%–97% among scrub typhus patients, depending on study site endemicity and study design [42,69]. It is important to realise that although the presence of an eschar is helpful, many scrub typhus patients may not have an eschar. Clearly, eschars are not helpful in a setting where eschars are found in as few as 7% among children, like in southern Thailand [70] or where the occurrence of eschars is at 18.2% among patients aged 13 years or older in Vietnam [37]. The prevalence of eschars also depends on the selection criteria of studies–if a study is centered around eschar presence as an inclusion criterion, a high eschar rate is likely to be found. In this retrospective study a high presence of eschars in scrub typhus patients with 93.1% in the 2013–2014 cohort was seen, but only in 52.6% in the 2018–2019 cohort. It is likely that in the first phase, doctors diagnosed scrub typhus based on eschars leading to a high eschar rate, while in 2018–2019, after our training of the medical staff, the awareness about scrub typhus cases without eschar was raised and additional improved diagnostics were introduced (ELISA assays diagnostics improved, thus leading to a lower eschar prevalence than before. It is important to consider that patients with spotted fever group rickettsioses may also present with eschars, and although extremely rare, a local lesion has been described in murine typhus [8,37,70–72], so the “pathognomonic” role of eschar in scrub typhus diagnosis should be considered carefully. Eschars usually present within 30 cm below the umbilicus (including the perineal, inguinal, and buttock areas), under the breasts in female patients and in the axillae/under upper skinfold of umbilicus of children [69,72,73]. Patients are often not willing to reveal these body parts to doctors, if they are not specifically asked about this—often resulting in missed eschars due to incomplete examinations [73].

With all above reasons, the importance of the eschar in scrub typhus diagnosis should be critically considered and if no eschar is found–despite thorough examination–the remaining 6 predictors or the CART decision tree “without eschar” should be considered as predictive indicators.

### Decision-supporting predictors based on multivariable logistic regression vs. CART

The CART analyses including “eschar” (i.e. an eschar was found), revealed after pruning that developing the tree necessitated two predictors only: “eschar” and “days of fever on admission”, leading to an index of Youden of 0.911. The application of the regression model to the entire data set revealed an index of Youden of 0.932 and involved the predictors: i) increased days of fever on admission, ii) regional lymphadenopathy, iii) an occupation in nature, iv) increased neutrophil count, v) decreased ratio of N/L (neutrophils/lymphocytes), and vi) age over 40, in addition to “eschar”. When not using the “eschar” variable, i.e. when no eschar was found–the regression model involved “an occupation in nature” and “age over 40”, while the CART tree involved “platelet count ≥ 47 G/L” and “age over 28”–in addition to the same predictors: days of fever on admission, regional lymphadenopathy, neutrophil, lymphocytes. However, both models resulted in similar accuracy for identification of scrub typhus (index of Youden: 0.681 vs. 0.686, respectively).

The documentation of “increased days of fever on admission” plays an important role in predicting scrub typhus. Due to self-treatment or misdiagnosis in community health centers, scrub typhus patients were likely to visit hospitals later than dengue patients. This can have an effect on diagnostics due to disease dynamics being characterized by an early bacteremia (7–10 days) followed by the antibody response–necessitating PCR to be coupled with a serological test for complete coverage of the diagnostic window [31]. If “an occupation in nature”, having “regional lymphadenopathy”, increased “days of fever on admission”, and “age >40 years” were combined, the area under the curve (ROC) was 86.2%. If neutrophils and lymphocytes were added, the AUC increased to 91.2% (Table 2). After further inclusion of “eschar”, the AUC reached 98.5% (Fig 3A1), meaning that correct application of these predictors in this cohort can contribute substantially to a presumptive diagnosis without a diagnostic test.

In this study, we applied two different statistical approaches for deriving a model to discriminate between scrub typhus and dengue fever; i) multivariable logistic regression (M-LR) and ii) CART. As results from M-LR, a set of given seven predictors produced a slightly improved predictive performance when compared with CART analyses (the index of Youden from M-LR and CART were 0.932 vs 0.911, respectively). The higher index of Youden of the M-LR approach might be due to starting the model without “eschar” and correcting the predictions of this model to an ST-probability of 1 in patients with eschar. This gives other variables a chance to also enter the model while “eschar” overshadows all other variables in the development of the regression tree. However, when excluding the “eschar” variable, both models performed similarly (the index of Youden being 0.686 for M-LR vs 0.681 for CART). The decision tree approach has several advantages over the approach using logistic regression. The first and most important advantage of a decision tree is that the derived rules and subgroups of the tree are easy to understand and sequentially lead the clinician along the branches of the tree to the presumptive diagnosis proposed by the algorithm [62]. For example, starting from the second level of flowchart in Fig 4B, if a patient has days of fever on admission ≥ 5 days, chances are 74% that he has scrub typhus. After that, if he has a neutrophil count ≥ 2.4, this increases the probability to 90%. This probability is increased to 94%, if a platelet count ≥47 G/L is present. If the regression-based algorithm is programmed it can also be applied swiftly if the input data are available. But of course one will then first have to enter all values, which takes longer than just following the tree visually and decisions can also be obtained very fast.

### Translating the findings into the real-world setting

Given the high probabilities of these predictors to make a presumptive diagnosis, they hold potential to inform a preemptive treatment strategy aiming to reduce complications and mortality, while creating improved awareness of scrub typhus all along.

These findings will be useful for medical staff working in areas where dengue and scrub typhus are endemic diseases. A simple medical history, a clinical examination and routine blood tests are available at primary health care centers, and will contribute to discriminating a bacterial from a viral disease in the 684 district hospitals and 11.083 community health centers. Correct application could lead to improved cost-efficiency by reducing medical and non-medical costs for patients, less unnecessary patient referrals to hospitals. Scrub typhus is an easily treatable disease with doxycycline which is inexpensive, readily available at local pharmacy agents and has a favorable age profile [74,75].

However, the importance of these findings lies in that an improved interpretation of readily available clinical-laboratory information could accelerate diagnosis and improve empirical treatment strategies at the primary health care level. Mis-diagnosis can contribute to antibiotic overuse, which is a substantial problem in Vietnam. Since scrub typhus does not respond to broadband antimicrobials such as betalactams (especially the common derivative cephalosporins, which are widely used for undifferentiated febrile illnesses), a decision algorithm in distinguishing scrub typhus from dengue would inform medical staff to choose more adequate or targeted treatment strategies to reduce antibiotic overuse (i.e. doxycycline or macrolides)—especially at the primary health care level [2].

Thus, application of these simple predictors in the correct way holds potential to i) reduce the delay to treatment initiation; ii) inform on the use of an adequate antimicrobial, iii) shorten the disease course to reduce complications and fatality rates, as well as iv) improve the management of uncomplicated fevers and create better awareness of scrub typhus.

### Limitations of the study

The data generated in this study is based on a large cohort of scrub typhus and dengue fever patients, since these together represent the major current burden of undifferentiated febrile disease. The findings need to be re-evaluated with a cohort including other co-endemic febrile illnesses, once more systematic evidence on the causes of undifferentiated febrile illness (UFI) becomes available. Likely diseases could be leptospirosis, murine typhus, Q fever, spotted fever group rickettsiae (SFGR), and/or melioidosis. This study has some limitations. Firstly: this was a retrospective study and the collected data could hold inconsistencies; secondly: eschar was among the main criteria to define a scrub typhus suspected case in the 2013–2014 period thus could contribute to introducing a selection bias. To counteract this, we enrolled all other undifferentiated fevers (patients with long-lasting fever over 10 days/undifferentiated fever/used medicine to reduce fever without effect; dengue/malaria suspected cases with negative dengue/malaria test results) to minimize losing potential cases and reduce the eschar-positive patient proportion; thirdly: all of the confirmed scrub typhus were tested for dengue fever and malaria, and co-infections were not included, however maybe this was not documented in all the cases, and a prospective study would provide more reliable results. Co-infections of scrub typhus and other UFI such as leptospirosis, murine typhus, Q fever, SFGR, melioidosis are expected to be rare, a small chance for co-infections remains. fourthly: Investigations were limited to dengue, scrub typhus and malaria, while other endemic diseases were not considered (i.e. chikungunya, zika, lyme, Q fever, spotted fever group rickettsia (SFGR), leptospirosis, murine typhus, and/or melioidosis). However, epidemiological reports suggest that at present Vietnam is considered a low-risk area for chikungunya, zika, lyme, Q fever, and SFGR [8,66,76–79]. Leptospirosis and murine typhus have been reported as causes of UFI in Vietnam. Although both diseases do not associate with eschars, they respond to doxycycline as empirical therapy [8,66], and clinical mis-classification of these two diseases as ST has no major therapeutic consequences [80–82]. The presented algorithms might not reach the accuracy reported if applied to areas with different epidemiological characteristics (i.e. settings with different risk factor profiles as in more urban areas), and the algorithms may require adaptation if improvements in dengue/scrub typhus diagnostic procedures occur. Positive and negative predictive values of the models need updating if an incidence change of these diseases and the other UFI above occurs over time; and fifthly, increased liver enzymes (ALT, AST) were described in differentiating scrub typhus from dengue fever patients previously in Thailand (19). Although 67% of scrub typhus cases (148/221) and <1% dengue fever cases (32/387) had elevated liver enzyme findings upon admission, they were not statistically associated with any predictive power for disease differentiation upon admission in the multivariable logistic model of this case-control study, which included >3 times more patients than the previous report.

## Conclusion

Scrub typhus and dengue fever are common sympatric endemic diseases in Vietnam. Basic clinical findings and routine hematological blood laboratory tests were investigated to develop a predictor-based clinical decision algorithm. The provided information by this study supports medical staff in the often challenging clinical decision-process for differentiating bacterial scrub typhus from viral dengue infections. Application of these simple predictors (Table 1) holds potential to i) improve clinical suspicion of scrub typhus cases; ii) reduce the delay to treatment initiation; iii) inform on the use of an adequate antimicrobial, iv) shorten the disease course to reduce complications and fatality rates, as well as v) improve the management of uncomplicated fevers and create better awareness of scrub typhus.

## Supporting information

S1 Data Full dataset in STATA format. (DTA)

S2 Data Full dataset in XCEL format. (XLSX)
